# eHealth literacy and decisional conflict in patients with coronary heart disease: chain mediation of cyberchondria and psychological inflexibility and a latent profile analysis

**DOI:** 10.3389/fpubh.2026.1964166

**Published:** 2026-10-01

**Authors:** Yan Huang, Tianyu She, Pei Gao, Ping Zhang, Yuhan Cai, Guihua Zeng, Donglian Liu, Zhao Tang, Gang Wen

**Affiliations:** 1 Clinical Pharmacy Center, Nanfang Hospital, Southern Medical University, Guangzhou, Guangdong, China; 2 Department of Medical Imaging Function, Xi'an Electric Power Central Hospital, Xi'an, Shaanxi, China; 3 Operating Department, Zhumadian Central Hospital, Zhumadian, Henan, China; 4 Department of Emergency, Nanfang Hospital, Southern Medical University, Guangzhou, Guangdong, China; 5 Department of Emergency, Ganzhou People's Hospital, Ganzhou, Jiangxi, China; 6 Department of Cardiology, Nanfang Hospital, Southern Medical University, Guangzhou, Guangdong, China

**Keywords:** coronary heart disease, cyberchondria, decisional conflict, eHealth literacy, psychological inflexibility

## Abstract

**Background:**

Coronary heart disease (CHD) is the leading cause of cardiovascular mortality worldwide, and its management requires patients to make complex, preference-sensitive treatment decisions. Decisional conflict is common in this population and adversely affects treatment adherence and outcomes, yet its association with eHealth literacy and the underlying psychological mechanisms remain unclear.

**Objective:**

This study examined the association between eHealth literacy and decisional conflict in patients with CHD, tested the chain-mediating roles of cyberchondria and psychological inflexibility, and identified latent profiles of eHealth literacy.

**Methods:**

A cross-sectional design was employed, and patients with CHD were recruited from tertiary Grade A hospitals. The survey battery comprised the eHealth Literacy Scale, the Decisional Conflict Scale, the Cyberchondria Severity Scale, the psychological inflexibility subscale of the Multidimensional Psychological Flexibility Inventory, and requisite demographic items.

**Results:**

eHealth literacy was significantly and negatively associated with decisional conflict, and this association was mediated by cyberchondria and psychological inflexibility. From a person-centered perspective, two latent subgroups of eHealth literacy were identified: a high eHealth literacy profile and a low eHealth literacy profile. One-way analysis of variance indicated that the optimal latent profiles differed significantly in decisional conflict, cyberchondria, and psychological inflexibility.

**Conclusion:**

This study not only helps identify priority targets for interventions addressing decisional conflict in patients with CHD, but also elucidates the psychological pathways through which eHealth literacy shapes treatment decision-making, thereby informing heterogeneity-informed, tailored clinical interventions.

## 1 Introduction

Cardiovascular diseases (CVDs) are the leading cause of death worldwide, accounting for an estimated 17.9 million deaths each year, and approximately 32% of all global mortality (1, 2). Among CVDs, coronary heart disease (CHD) is the most common and most lethal condition; the Global Burden of Disease Study estimated that ischaemic heart disease was responsible for roughly 9.1 million deaths in 2021, making it the single largest contributor to global mortality (3, 4). The burden is especially pronounced in China, where more than 330 million people live with CVD and the prevalence of CHD exceeds 11 million, and figures that continue to rise with population aging and the adoption of Westernized lifestyles (5, 6). CHD therefore constitutes a global public health priority that places heavy demands on health systems and on patients’ capacity for long-term self-management.

Contemporary CHD treatment, which includes optimal medical therapy, percutaneous coronary intervention, and coronary artery bypass grafting, has markedly improved survival and quality of life (7, 8). Nevertheless, treatment is inherently complex and preference-sensitive: patients must weigh therapeutic benefit against procedural risk, financial cost, and personal values across a long-term, context-dependent illness trajectory (9, 10). When choosing among treatment options or making everyday decisions about medication adjustment, revascularization timing, follow-up scheduling, cardiac rehabilitation, and lifestyle change, many patients experience uncertainty, a perceived lack of information, unclear values, and insufficient support, the defining features of decisional conflict. Decisional conflict can delay treatment, impair physician–patient communication, reduce adherence, and worsen anxiety and depression, ultimately compromising outcomes (11, 12). Understanding its modifiable determinants is therefore essential for developing more precise decision-support interventions in clinical practice.

In the digital era, online health information has become a important channel through which patients with CHD acquire disease knowledge and evaluate treatment options (13, 14). Yet access to information does not guarantee its effective use: the extent to which patients can seek, understand, appraise, and apply electronic health information varies widely and may shape both the quality of treatment decisions and the psychological processes underlying them (15, 16). The present study therefore investigates how eHealth literacy is associated with decisional conflict in patients with CHD and through which psychological mechanisms.

## 2 Background

### 2.1 eHealth literacy and decisional conflict

eHealth literacy is defined as the ability to seek, find, understand, and appraise health information from electronic sources and apply the resulting knowledge to address a health problem (17). In patients with CHD, higher eHealth literacy has been associated with better identification of authoritative sources, improved comprehension of pharmacotherapy and interventional procedures, and more targeted questioning during clinical encounters, which may enhance participation in decision-making (18, 19). Conversely, inadequate eHealth literacy leaves patients vulnerable to one-sided, emotionally charged, or commercially driven content that can amplify perceived disease severity and treatment risk. Importantly, greater information exposure does not necessarily improve decision quality: without critical appraisal, redundant or conflicting information can increase cognitive load and choice difficulty (20, 21). In the digital health environment, patients with CHD may therefore paradoxically experience greater decisional uncertainty despite access to abundant information—a possibility that has received limited empirical attention.

### 2.2 Cyberchondria

Cyberchondria refers to the escalation of health anxiety driven by repeated or excessive online searching for health information (22). Its core features, which includes catastrophic interpretation of bodily symptoms, repeated reassurance-seeking, and selective attention to risk cues (23, 24), are highly relevant to CHD, in which ambiguous somatic signals such as chest pain, palpitations, and dyspnea are common (25). When eHealth literacy is inadequate, patients who encounter online content about myocardial infarction, in-stent restenosis, or adverse drug reactions may be unable to discriminate valid from invalid information, producing exaggerated threat perception and driving a self-perpetuating cycle of searching and anxiety (26). This cycle may erode trust in evidence-based recommendations and intensify hesitation and conflict regarding treatment choices.

### 2.3 Psychological inflexibility

Psychological inflexibility denotes the tendency to respond to distressing or uncertain experiences with experiential avoidance, cognitive fusion, and rigid behavioral patterns (27). Treatment decision-making in CHD requires confronting probabilistic risk, the burden of long-term medication, and the psychological strain of lifestyle change (28). Individuals high in psychological inflexibility tend to treat worrisome thoughts as facts, interpret transient somatic discomfort as serious deterioration, and reduce distress by avoiding decisions, delaying treatment, or seeking reassurance, and strategies that relieve distress only transiently and ultimately reinforce decisional difficulty.

### 2.4 Theoretical framework

We integrate three complementary theoretical perspectives to specify how eHealth literacy may shape decisional conflict in patients with CHD. First, the Ottawa Decision Support Framework conceptualizes decisional conflict as arising from perceived informational inadequacy, unclear values, and inadequate support (29, 30); within this framework, eHealth literacy is a proximal determinant of the informational and value-clarity prerequisites of high-quality decisions. Second, the cognitive-behavioral model of cyberchondria (31) explains how information-appraisal deficits translate into health anxiety: low eHealth literacy impairs the filtering and critical appraisal of online content, so that ambiguous or alarming information is more readily interpreted as threatening, initiating the searching–anxiety cycle. Third, the psychological flexibility model underpinning Acceptance and Commitment Therapy (32, 33) explains how health anxiety becomes entrenched as decisional avoidance: the threat orientation engendered by cyberchondria fosters cognitive fusion and experiential avoidance, predisposing patients to escape from, rather than actively engage with, decisional situations.

These perspectives imply a serial pathway in which low eHealth literacy increases cyberchondria, which in turn heightens psychological inflexibility and thereby intensifies decisional conflict. We acknowledge, however, that the relation between cyberchondria and psychological inflexibility is plausibly reciprocal rather than strictly unidirectional. On the one hand, the health anxiety and repetitive reassurance-seeking characteristic of cyberchondria may reinforce cognitive fusion and experiential avoidance (34); on the other hand, a pre-existing rigid, avoidant regulatory style may dispose patients toward catastrophic interpretation and compulsive searching, such that psychological inflexibility may in some circumstances precede and amplify cyberchondria. Our cross-sectional model specifies the health-anxiety-to-avoidance ordering (cyberchondria → psychological inflexibility) on theoretical and empirical grounds, but we treat the possibility of reverse and bidirectional effects as an explicit limitation and a focus for longitudinal testing.

### 2.5 The present study

Despite a growing literature, few studies have integrated eHealth literacy, cyberchondria, psychological inflexibility, and decisional conflict within a single framework in patients with CHD, and conventional variable-centered analyses cannot reveal how these constructs combine within individuals. The purpose of the present study was therefore twofold: (1) to examine whether cyberchondria and psychological inflexibility serially mediate the association between eHealth literacy and decisional conflict in patients with CHD (a variable-centered approach); and (2) to identify latent profiles of eHealth literacy in this population and compare the resulting profiles on cyberchondria, psychological inflexibility, and decisional conflict (a person-centered approach). We addressed the following research questions:

*RQ1*: Is eHealth literacy significantly and negatively associated with decisional conflict in patients with CHD?

*RQ2*: Does cyberchondria mediate the association between eHealth literacy and decisional conflict?

*RQ3*: Does psychological inflexibility mediate the association between eHealth literacy and decisional conflict?

*RQ4*: Do cyberchondria and psychological inflexibility serially (chain) mediate the association between eHealth literacy and decisional conflict?

*RQ5*: Is there latent heterogeneity in eHealth literacy among patients with CHD?

*RQ6*: Do the optimal latent profiles differ significantly in cyberchondria, psychological inflexibility, and decisional conflict?

## 3 Methods

### 3.1 Study design

This study adopted a descriptive, cross-sectional, analytical design to examine the associations among eHealth literacy, cyberchondria, psychological inflexibility, and decisional conflict (35, 36). The study protocol was reviewed and approved by the Medical Ethics Committee of Zhumadian Central Hospital (No.: 2026-04-KY007). All participants were fully informed of the study purpose, content, and potential risks and benefits, and voluntarily provided written informed consent. To minimize response bias, questionnaires were completed anonymously, and participants were explicitly told that there were no right or wrong answers and that their responses would remain confidential.

### 3.2 Participants

Patients with CHD were recruited from the cardiology departments of four hospitals in China between April and June 2026 using convenience sampling (37). The four sites were selected to capture geographic and socioeconomic diversity: (1) Nanfang Hospital, Southern Medical University, a large university-affiliated tertiary teaching hospital in Guangzhou, Guangdong Province, southern China; (2) Xi’an Electric Power Central Hospital, a category A general hospital of class II in Xi’an, Shaanxi Province, northwestern China; (3) Zhumadian Central Hospital, a tertiary general hospital in Zhumadian, Henan Province, central China; and (4) Ganzhou People’s Hospital, a tertiary general hospital in Ganzhou, Jiangxi Province, southeastern China. Together, these sites span four provinces across southern, central, northwestern, and southeastern China, thereby broadening the geographic and socioeconomic representativeness of the sample and supporting the international generalizability of the findings.

Specifically, the research team first developed an electronic questionnaire and a standardized paper-based questionnaire on a professional data collection platform and generated a corresponding QR code. The team then contacted the directors of the cardiology departments of the participating hospitals to explain the study objectives, procedures, and potential risks in detail; data collection commenced after approval was obtained. Before questionnaire administration, patients were fully informed about the risks, purpose, benefits, and intended data use of the survey. Only patients who provided written informed consent proceeded to complete the questionnaire. Patients able to use mobile electronic devices completed the electronic version, whereas the remaining patients completed the paper-based version.

To enhance the validity of the findings, strict eligibility criteria were applied. Inclusion criteria were: (1) age ≥ 18 years; (2) a definite diagnosis of CHD based on coronary angiography, coronary computed tomography angiography, a documented history of myocardial infarction, prior PCI or CABG, or diagnosis by a cardiovascular specialist in accordance with established diagnostic criteria; (3) clinically stable condition permitting questionnaire completion during the survey period; (4) basic Chinese-language communication and reading comprehension ability; and (5) willingness to participate and provision of written informed consent.

Exclusion criteria were: (1) severe cognitive impairment, disturbance of consciousness, psychotic disorder, or any other condition precluding comprehension of the questionnaire; (2) acute critical illness, such as the acute phase of myocardial infarction, decompensated severe heart failure, or unstable malignant arrhythmia; (3) severe comorbid physical illness rendering questionnaire completion unfeasible, such as end-stage malignancy or severe hepatic or renal failure; and (4) refusal to participate or withdrawal during the survey.

The sample size was determined *a priori* on the basis of two considerations. First, for the serial mediation model, we used the empirical power tables of Fritz and Mackinnon (38) for the bias-corrected bootstrap test of the indirect effect; assuming small-to-medium standardized path coefficients (*a* and *b* ≈ 0.26) and targeting 80% statistical power at a two-tailed α of 0.05, a minimum of approximately 400 participants was required. Second, for latent profile analysis, a minimum of 300 participants is recommended to ensure stable class enumeration and adequate classification accuracy (39). Accounting for an anticipated ~10% rate of invalid questionnaires, we aimed to enroll approximately 450 patients. During recruitment, 436 patients were approached; of these, 14 had not yet received a confirmed diagnosis of CHD and 19 provided regular patterned responses, yielding a final analytic sample of 403 patients and an effective response rate of 92.43%, which satisfied the sample size requirement.

### 3.3 Measures tools

#### 3.3.1 eHealth literacy scale

eHealth literacy was assessed using the simplified Chinese version of the eHealth Literacy Scale (eHEALS) adapted by Xu et al. (40). This unidimensional instrument comprises eight items (e.g., “I know what health resources are available on the Internet”) and has been widely applied in patient populations (41, 42). Items were rated on a 5-point Likert scale ranging from 1 (“strongly disagree”) to 5 (“strongly agree”). The scale contains no reverse-scored items; total scores range from 8 to 40, with higher scores indicating higher self-perceived eHealth literacy. In the present study, Cronbach’s α for this scale was 0.913, indicating good internal consistency.

#### 3.3.2 Decisional conflict scale

Decisional conflict was assessed using the Mandarin version of the statement-format Decisional Conflict Scale adapted by Lu et al. (43). The scale comprises 16 items across five dimensions: Informed, Values Clarity, Support, Uncertainty, and Effective Decision (e.g., “I am clear about which risks and side effects matter most to me”). Items were rated on a 5-point Likert scale ranging from 1 (“strongly disagree”) to 5 (“strongly agree”). The scale contains no reverse-scored items; total scores range from 16 to 80, with higher scores indicating greater decisional conflict when facing treatment decisions. In the present study, Cronbach’s α was 0.931, indicating good internal consistency.

#### 3.3.3 Cyberchondria severity scale

Cyberchondria was assessed using the Chinese version of the Cyberchondria Severity Scale adapted by Wang et al. (44). The scale comprises 18 items across three dimensions—negative effects, excessiveness, and reassurance seeking (e.g., “Researching symptoms or perceived medical conditions online interrupts my online leisure activities, such as watching streaming movies”). Items were rated on a 5-point Likert scale ranging from 1 (“never”) to 5 (“always”). The scale contains no reverse-scored items; total scores range from 18 to 90, with higher scores indicating greater cyberchondria severity. In the present study, Cronbach’s α was 0.913, indicating good internal consistency.

#### 3.3.4 Psychological inflexibility scale

Psychological inflexibility was assessed using the psychological inflexibility component of the Multidimensional Psychological Flexibility Inventory (MPFI) developed by Rolffs et al. (45). The MPFI comprises parallel flexibility and inflexibility components; the present study administered the 12-item inflexibility component, which covers six dimensions: Lack of Contact with the Present Moment, Experiential Avoidance, Fusion, Self as Content, Inaction, and Lack of Contact with Values (e.g., “I found myself doing things without paying much attention”). The Chinese version was adapted by Liu et al. (46), who verified its cultural appropriateness and reliability in Chinese samples. Items were rated on a 5-point Likert scale ranging from 1 (“never true”) to 5 (“always true”). The scale contains no reverse-scored items; total scores range from 12 to 60, with higher scores indicating greater psychological inflexibility. In the present study, Cronbach’s α was 0.887, indicating good internal consistency.

### 3.4 Statistical analysis

All analyses were conducted using SPSS 27.0 and Mplus 8.3. The analytic sequence, organized to correspond to the research questions, was as follows:

Preliminary analyses: descriptive statistics, internal-consistency reliability (Cronbach’s α), Harman’s single-factor test for common method bias, and bivariate correlations among the focal variables.

Between-group comparisons: independent-samples t tests and one-way analyses of variance examined differences in decisional conflict across demographic and disease-related characteristics to select covariates for the mediation model.

Serial mediation (RQ1–RQ4): PROCESS Model 6 was used to test the cross-sectional chain mediation of cyberchondria and psychological inflexibility linking eHealth literacy to decisional conflict, with sex, marital status, and history of cardiac intervention or surgery entered as covariates. Indirect effects were tested using the bias-corrected bootstrap method with 5,000 resamples, with significance inferred when the 95% confidence interval excluded zero.

Latent profile analysis (RQ5): LPA was conducted with the eight eHEALS items as indicators; models specifying one to five classes were fitted sequentially, and the optimal solution was determined by a combination of fit indices (AIC, BIC, aBIC, entropy, Lo–Mendell–Rubin test, bootstrap likelihood ratio test) and substantive interpretability.

Profile comparisons (RQ6): independent-samples t tests compared the optimal latent profiles on cyberchondria, psychological inflexibility, and decisional conflict.

## 4 Results

### 4.1 Sample characteristics

A total of 403 patients with CHD were included, with a mean age of 67.35 ± 13.467 years. Of these, 210 (52.1%) were male and 193 (47.9%) were female. Overall, 168 participants (41.7%) had a middle school – high school education, 309 (76.7%) were married, and 272 (67.5%) were urban residents. Detailed demographic characteristics are presented in Table 1.

**Table 1:** Demographic characteristics of participants.

Variables	Items	Number (N)	Proportion (%)
Gender	Male	210	52.1%
	Female	193	47.9%
Educational attainment	Primary school and below	155	38.5%
	Middle school – High school	168	41.7%
	Bachelor degree or above	80	19.9%
Marital status	Married	309	76.7%
	Unmarried	31	7.7%
	Divorced	24	6.0%
	Widowed spouse	39	9.7%
Place of residence	Urban	272	67.5%
	Rural	131	32.5%
Employment status	On the job	128	31.8%
	Retirement	229	56.8%
	Unemployment	46	11.4%
Individual monthly income	Less than 4,000 CNY	130	32.3%
	4,001–8,000 CNY	184	45.7%
	≥8,001 CNY	89	22.1%
Time since CHD diagnosis	<1 year	82	20.3%
	1–5 years	146	36.2%
	5–10 years	117	29.0%
	>10 years	58	14.4%
History of cardiac intervention or surgery	Yes	146	36.2%
	No	257	63.8%
Living arrangement	Live alone	110	27.3%
	Live with your spouse	200	49.6%
	Live with your children	57	14.1%
	Nursing home	36	8.9%
Age	*M* ± SD	67.35 ± 13.467	

### 4.2 Common method bias test

Procedural remedies, including confidentiality assurances, anonymous administration, and explicit statements that there were no right or wrong answers, were implemented to reduce common method bias. Nonetheless, because all focal variables were assessed via self-report, the risk of common method bias could not be eliminated entirely.

Harman’s single-factor test was therefore conducted as a preliminary assessment. Unrotated exploratory factor analysis extracted five factors with eigenvalues greater than 1, and the first factor accounted for 29.400% of the total variance, below the 40% criterion. These results suggest that severe common method bias is unlikely in the present data.

### 4.3 Descriptive statistics and correlation analysis

Descriptive statistics and bivariate correlations for the focal variables are presented in Table 2. Overall, eHealth literacy was relatively low (2.212 ± 0.868), whereas cyberchondria (3.746 ± 0.722), psychological inflexibility (3.737 ± 0.753), and decisional conflict (3.797 ± 0.763) were relatively high, suggesting that this population may experience considerable psychological burden and decisional difficulty with respect to digital health information processing, health anxiety responses, psychological regulatory flexibility, and treatment decision-making.

**Table 2:** Descriptive statistics and correlation analysis of core study variables.

Variables	*M*	SD	Skewness	Kurtosis	1	2	3	4
1. eHealth literacy	2.212	0.868	1.494	0.971	1			
2. Cyberchondria	3.746	0.722	−1.406	0.939	−0.491**	1		
3. Psychological inflexibility	3.737	0.753	−1.375	0.871	−0.387**	0.626**	1	
4. Decisional conflict	3.797	0.763	−1.533	0.885	−0.344**	0.507**	0.492**	1

**p < 0.01.

Correlation analyses indicated that eHealth literacy was significantly negatively correlated with cyberchondria (*r* = −0.491, *p* < 0.01), psychological inflexibility (r = −0.387, *p* < 0.01), and decisional conflict (*r* = −0.344, *p* < 0.01). In addition, cyberchondria was significantly positively correlated with psychological inflexibility (*r* = 0.626, *p* < 0.01) and decisional conflict (*r* = 0.507, *p* < 0.01), and psychological inflexibility was significantly positively correlated with decisional conflict (*r* = 0.492, *p* < 0.01).

According to the criteria proposed by Kline (47), absolute skewness values for the focal variables ranged from 1.375 to 1.533 and absolute kurtosis values ranged from 0.871 to 0.971, indicating approximate normality.

### 4.4 Differences in decisional conflict across demographic characteristics

Differences in decisional conflict across demographic and disease-related characteristics are presented in Table 3. Significant differences were observed for sex (*t* = −2.22, *p* = 0.027), marital status (*F* = 3.547, *p* = 0.015), and history of cardiac intervention or surgery (*t* = −2.842, *p* = 0.005). By contrast, no significant differences were found for educational attainment, place of residence, employment status, monthly personal income, time since CHD diagnosis, or living arrangement (*p* > 0.05).

**Table 3:** Between-group comparisons of decisional conflict across demographic characteristics.

Variables	Items	*M*	SD	*F*/*t*	*p*	Cohen *d*/*η*^2^
Gender	Male	3.717	0.837	−2.22	0.027	−0.221
	Female	3.885	0.663			
Educational background	Primary school and below	3.788	0.752	0.053	0.948	0.001
	Middle school – High school	3.794	0.759			
	Bachelor degree or above	3.822	0.797			
Marital status	Married	3.814	0.757	3.547	0.015	0.026
	Unmarried	4.038	0.507			
	Divorced	3.810	0.704			
	Widowed spouse	3.470	0.918			
Place of residence	Urban	3.758	0.791	−1.497	0.135	−0.159
	Rural	3.879	0.695			
Employment status	On the job	3.791	0.746	0.778	0.460	0.004
	Retirement	3.775	0.785			
	Unemployment	3.928	0.694			
Individual monthly income	Less than 4,000 yuan	3.717	0.822	1.078	0.341	0.005
	4,001–8,000 yuan	3.833	0.722			
	≥8,001 yuan	3.843	0.754			
Duration of diagnosis of coronary heart disease	<1 year	3.832	0.715	0.200	0.897	0.001
	1–5 years	3.818	0.782			
	5–10 years	3.763	0.761			
	>10 years	3.767	0.799			
Previous history of cardiac intervention/surgery	Yes	3.655	0.866	−2.842	0.005	−0.295
	No.	3.878	0.686			
Mode of living	Live alone	3.780	0.775	0.038	0.990	0.001
	Live with your spouse	3.800	0.758			
	Live with your children	3.821	0.800			
	Nursing home	3.799	0.721			

### 4.5 Cross-sectional chain mediation of cyberchondria and psychological inflexibility

PROCESS Model 6 was used to construct a cross-sectional serial mediation model, with eHealth literacy as the independent variable, cyberchondria and psychological inflexibility as sequential mediators, and decisional conflict as the dependent variable; sex, marital status, and history of cardiac intervention or surgery were entered as covariates (Table 4 and Figure 1).

**Table 4:** Regression analyses for cyberchondria and psychological inflexibility.

Model	Model 1			Model 2			Model 3		
Predictor	Cyberchondria			Psychological inflexibility			Decisional conflict		
	β	t	95%CI	β	t	95%CI	β	t	95%CI
eHealth literacy	−0.491	−11.289***	[−0.479,-0.338]	−0.105	−2.3701*	[−0.167,-0.016]	−0.096	−2.0084*	[−0.168,-0.002]
Cyberchondria				0.574	12.9217***	[0.507,0.689]	0.288	5.072***	[0.186, 0.421]
Psychological inflexibility							0.274	5.1212***	[0.171, 0.385]
*R*	0.491			0.633			0.560		
*R* ^2^	0.241			0.400			0.314		
*F*	127.445***			133.540***			60.763***		

***p < 0.001, *p < 0.05.

**Figure 1:**
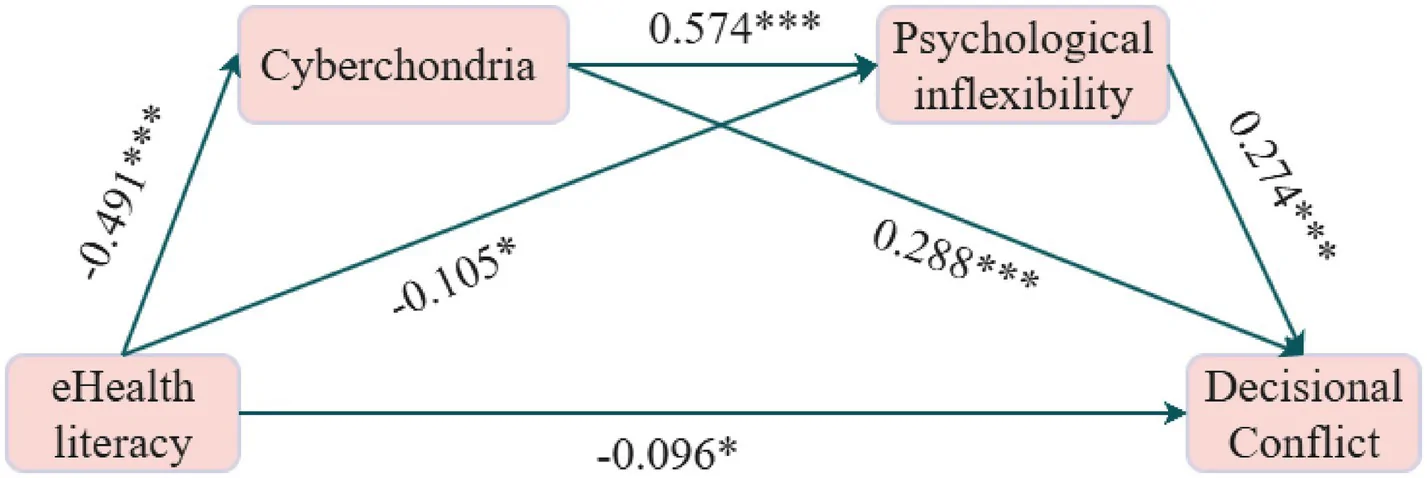
Path coefficients of the cross-sectional chain mediation model involving cyberchondria and psychological inflexibility. ***p < 0.001, *p < 0.05.

In Model 1, with cyberchondria as the outcome, eHealth literacy significantly negatively predicted cyberchondria (*β* = −0.491, *p* < 0.001, 95% CI = [−0.479, −0.338]). This model accounted for 24.1% of the variance in cyberchondria (*R*^2^ = 0.241, *F* = 127.445).

In Model 2, with psychological inflexibility as the outcome, eHealth literacy remained a significant negative predictor (*β* = −0.105, *p* < 0.05, 95% CI = [−0.167, −0.016]), while cyberchondria significantly positively predicted psychological inflexibility (*β* = 0.574, *p* < 0.001, 95% CI = [0.507, 0.689]). This model accounted for 40.0% of the variance in psychological inflexibility (*R*^2^ = 0.400, *F* = 133.540).

In Model 3, with decisional conflict as the outcome, eHealth literacy remained a significant negative predictor (*β* = −0.096, *p* < 0.05, 95% CI = [−0.168, −0.002]); cyberchondria significantly positively predicted decisional conflict (*β* = 0.288, *p* < 0.001, 95% CI = [0.186, 0.421]); and psychological inflexibility likewise significantly positively predicted decisional conflict (*β* = 0.274, *p* < 0.001, 95% CI = [0.171, 0.385]). This model accounted for 31.4% of the variance in decisional conflict (*R*^2^ = 0.314, *F* = 60.763).

Mediating effects were tested using the bias-corrected bootstrap method with 5,000 resamples (Table 5). The total effect of eHealth literacy on decisional conflict was significant (Effect = −0.302, SE = 0.041, 95% CI = [−0.383, −0.221]). After including cyberchondria and psychological inflexibility, the direct effect of eHealth literacy on decisional conflict remained significant (Effect = −0.085, SE = 0.042, 95% CI = [−0.168, −0.002]), accounting for 28.00% of the total effect; RQ1 was therefore supported.

**Table 5:** Decomposition of the cross-sectional chain mediation effects of cyberchondria and psychological inflexibility.

Type of effect	Effect	SE	LLCI	ULCI	Proportion of effect	Support hypothesis
Total effect	−0.302	0.041	−0.383	−0.221	100.00%	
Direct effect	−0.085	0.042	−0.168	−0.002	28.00%	RQ1
Indirect effect	−0.218	0.039	−0.296	−0.145	72.00%	
Ind 1	−0.124	0.034	−0.195	−0.065	41.08%	RQ2
Ind 2	−0.025	0.014	−0.055	−0.001	8.41%	RQ3
Ind 3	−0.068	0.017	−0.105	−0.037	22.51%	RQ4

The overall cross-sectional indirect effect through cyberchondria and psychological inflexibility was significant (Effect = −0.218, SE = 0.039, 95% CI = [−0.296, −0.145]), accounting for 72.00% of the total effect. The first indirect pathway, “eHealth literacy → cyberchondria → decisional conflict,” yielded an effect of −0.124 (SE = 0.034, 95% CI = [−0.195, −0.065]), representing 41.08% of the total effect and supporting RQ2. The second pathway, “eHealth literacy → psychological inflexibility → decisional conflict,” yielded an effect of −0.025 (SE = 0.014, 95% CI = [−0.055, −0.001]), representing 8.41% of the total effect and supporting RQ3. The third pathway, “eHealth literacy → cyberchondria → psychological inflexibility → decisional conflict,” yielded an effect of −0.068 (SE = 0.017, 95% CI = [−0.105, −0.037]), representing 22.51% of the total effect and supporting RQ4.

Taken together, these findings suggest that inadequate eHealth literacy may first heighten cyberchondria, such that exposure to online health information generates greater perceived health threat and repeated reassurance-seeking; cyberchondria in turn strengthens psychological inflexibility, predisposing patients toward cognitive fusion, experiential avoidance, and decisional avoidance, and ultimately exacerbating decisional conflict.

### 4.6 Latent profile analysis of eHealth literacy in patients with CHD

To identify latent heterogeneity in eHealth literacy from a person-centered perspective, latent profile analysis was conducted using the eHEALS items as indicators, fitting one- to five-class models sequentially; fit indices are presented in Table 6.

**Table 6:** Fit indices for the one- to five-class latent profile models of eHealth literacy in patients with CHD.

Index	Profile 1	Profile 2	Profile 3	Profile 4	Profile 5
AIC	9791.371	7627.587	7571.519	7544.271	7524.192
BIC	9855.354	7727.560	7707.483	7716.225	7732.137
aBIC	9804.584	7648.233	7599.597	7579.782	7567.135
Entropy	–	0.997	0.980	0.777	0.808
LMR (p)	–	< 0.001	0.099	0.273	0.770
BLRT (p)	–	< 0.001	< 0.001	< 0.001	< 0.001
Class 1	–	81.89%	81.89%	53.60%	31.02%
Class 2	–	18.11%	5.21%	28.29%	49.38%
Class 3	–	–	12.90%	13.15%	2.98%
Class 4	–	–	–	4.96%	11.91%
Class 5	–	–	–	–	4.72%

As the number of classes increased from one to five, AIC, BIC, and aBIC generally decreased, indicating improved model fit with additional classes. The two-class model showed markedly lower AIC, BIC, and aBIC than the one-class model, with exceptionally high classification accuracy (entropy = 0.997); both the LMR test (*p* < 0.001) and the BLRT (*p* < 0.001) were significant, indicating that the two-class solution fit significantly better than the one-class solution. When the three-class model was further compared, the LMR test did not reach statistical significance (*p* = 0.099), indicating that the three-class solution did not provide a substantive improvement over the two-class solution. Although AIC continued to decrease for the four- and five-class models, entropy declined appreciably, the LMR tests were non-significant, and the five-class solution contained a small class comprising only 2.98% of the sample, suggesting a risk of over-extraction.

Accordingly, the two-class model was retained as the optimal latent profile solution for eHealth literacy in patients with CHD, supporting RQ5.

To further evaluate classification quality, average posterior probabilities for the two-class solution were computed (Table 7). The average posterior probability of correct assignment was 0.999 for Class 1 and 1.000 for Class 2. The two-class model thus demonstrated excellent classification precision and class separation, with clear boundaries between latent classes and reliable classification results.

**Table 7:** Average posterior probabilities for the optimal latent profile solution.

Class	Class 1	Class 2
Class 1	0.999	0.001
Class 2	0.000	1.000

Based on the item-level mean profiles and class characteristics (Figure 2), Class 1 was labeled the “low eHealth literacy group,” comprising 81.89% of the sample, and Class 2 the “high eHealth literacy group,” comprising 18.11%. The class proportions indicate that most patients with CHD fall into the low eHealth literacy group, suggesting widespread deficits in the ability to seek, understand, appraise, and apply online health information within this population.

**Figure 2:**
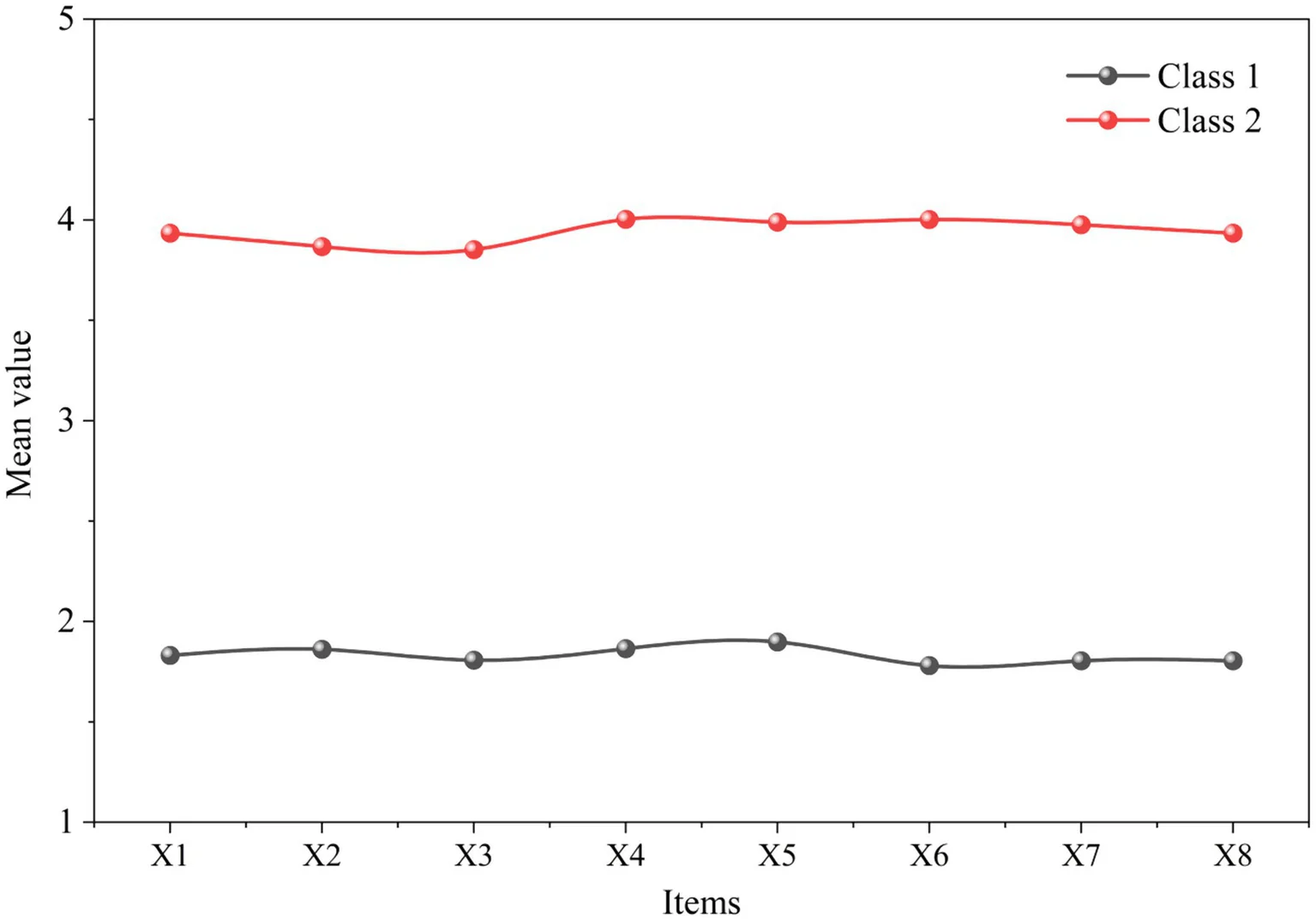
Profile plot of the optimal latent profile solution. Class 1 = low eHealth literacy group; Class 2 = high eHealth literacy group.

### 4.7 Univariate comparisons across the optimal latent profiles

To examine differences between eHealth literacy profiles in cyberchondria, psychological inflexibility, and decisional conflict, independent-samples t tests were conducted (Table 8 and Figure 3), supporting RQ6. Specifically, the two profiles differed significantly in cyberchondria (*t* = 12.921, *p* < 0.001), psychological inflexibility (*t* = 8.747, *p* < 0.001), and decisional conflict (*t* = 7.718, *p* < 0.001).

**Table 8:** Differences between the optimal latent profiles in cyberchondria, psychological inflexibility, and decisional conflict.

Variables	Class	*M*	SD	*t*	*p*	Cohen *d*
Cyberchondria	Class 1	3.929	0.538	12.921	<0.001	1.671
	Class 2	2.914	0.857			
Psychological inflexibility	Class 1	3.878	0.594	8.747	<0.001	1.131
	Class 2	3.097	1.020			
Decisional conflict	Class 1	3.926	0.631	7.718	<0.001	0.998
	Class 2	3.215	1.005			

**Figure 3:**
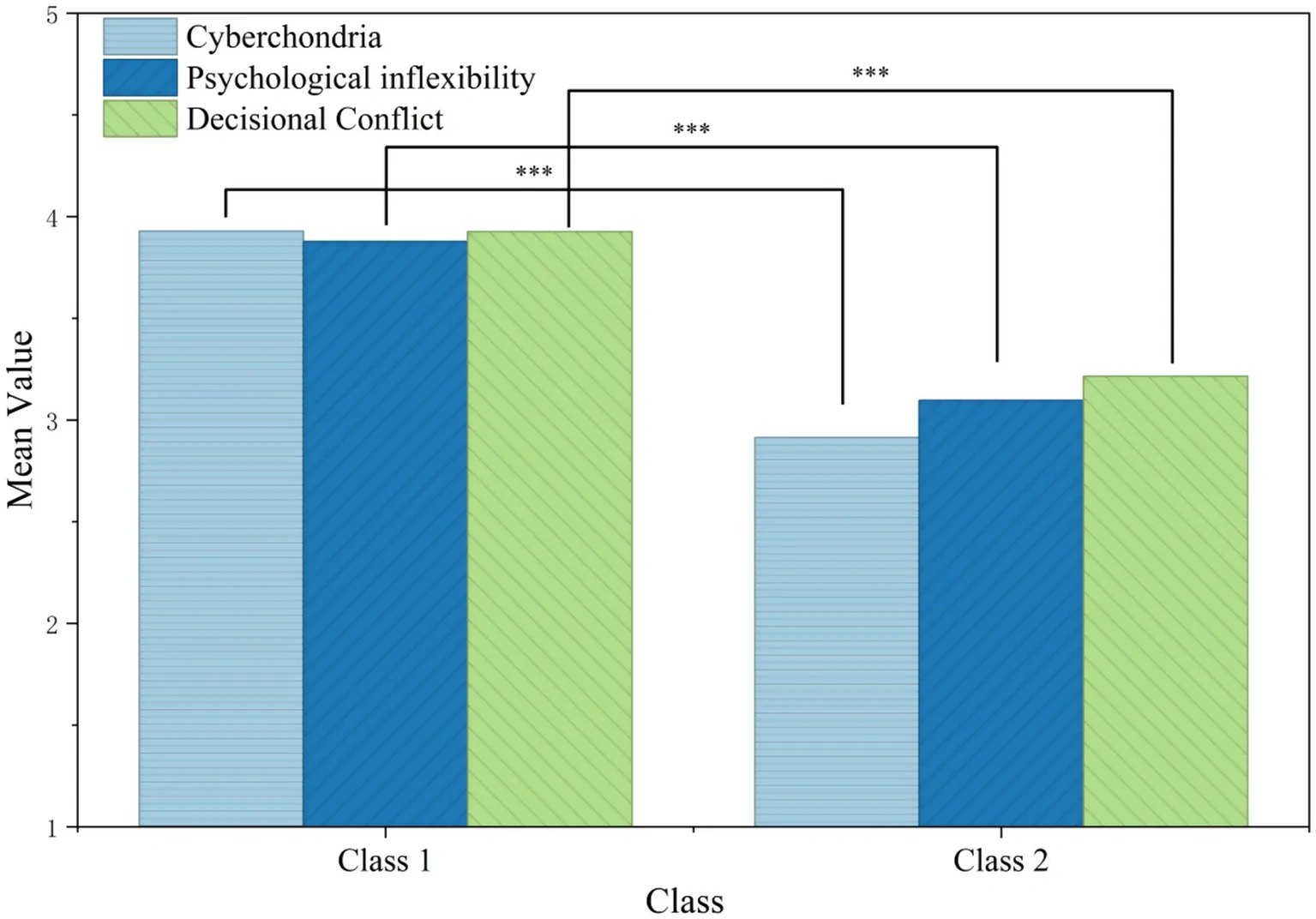
Difference comparison of optimal latent profile in cyberchondria, psychological inflexibility and treatment decision conflict. Class 1 = low eHealth literacy group; Class 2 = high eHealth literacy group.

## 5 Discussion

### 5.1 Variable-centered perspective: cross-sectional mediation by cyberchondria and psychological inflexibility

Consistent with the protective role of health literacy in chronic-disease self-management (48, 49), eHealth literacy was negatively associated with decisional conflict in this cohort of patients with CHD. Rather than restating the magnitude of this association, we emphasize what it means for this specific population. Because CHD management is characterized by long-term medication, repeated procedural decisions, and lifestyle restructuring, patients are continuously confronted with probabilistic risk information (50, 51). Those with adequate eHealth literacy can discriminate authoritative guidance from unvetted content and therefore form clearer value judgments before high-stakes choices (52), whereas those with low literacy are more exposed to online risk narratives, such as accounts of stent thrombosis, in-stent restenosis, or statin side effects, that are disproportionately salient for CHD. The marked contrast between the low mean eHealth literacy and the relatively high decisional conflict observed in this older, predominantly low-education sample indicates substantial room to reduce decisional difficulty by strengthening digital health competence.

Cyberchondria was the strongest mediator of the eHealth literacy–decisional conflict association. This finding is particularly meaningful in CHD because the cardinal symptoms of this disease, which includes chest discomfort, dyspnea, palpitations, and fatigue (53), are intrinsically ambiguous and overlapping with benign conditions, making them fertile ground for catastrophic online self-diagnosis. For patients who have undergone PCI or CABG, the fear of graft failure or stent occlusion is concrete, easily searchable, and frequently amplified by non-authoritative sources; reassurance-seeking therefore becomes both understandable and self-defeating, trapping patients in a searching–anxiety loop that directly undermines confidence in treatment choices. This mechanism is likely more pronounced in CHD than in many other chronic conditions precisely because the perceived stakes (cardiac events) are perceived as immediate and life-threatening.

Psychological inflexibility was a comparatively weaker mediator, which we interpret in light of its status as a relatively stable, trait-like disposition shaped by long-term emotional-regulation history rather than by competence in any single domain. Nevertheless, its significance is clinically relevant for CHD: the need to accept irreducible prognostic uncertainty (e.g., residual cardiovascular risk despite optimal therapy) is a distinctive psychological demand of this disease. Patients who fuse with catastrophic thoughts and avoid the discomfort of uncertainty may postpone revascularization or refuse recommended therapy, converting an informational deficit into a behavioral impasse.

The serial pathway illustrates how, in CHD, an informational deficit can be transformed into a self-reinforcing psychological cascade. Persistent health anxiety progressively erodes psychological flexibility, so that patients increasingly disengage from active deliberation and instead oscillate between reassurance-seeking and avoidance. The strong cyberchondria–psychological inflexibility association observed here underscores that, in this population, health anxiety and psychological rigidity frequently co-occur and mutually reinforce one another—consistent with the reciprocal relation we articulated in the theoretical framework.

### 5.2 Person-centered perspective: heterogeneity in eHealth literacy

Latent profile analysis further revealed underlying heterogeneity in eHealth literacy among patients with CHD, yielding a “low eHealth literacy group” and a “high eHealth literacy group.” eHealth literacy is thus not homogeneously distributed but instead differentiates into distinct latent classes. Moreover, most patients fell into the low eHealth literacy class, indicating that CHD populations may have pervasive deficits in using digital health resources, appraising online information, and translating health information into action.

Disease management for patients with CHD is increasingly extending from traditional offline health care settings into digital environments. However, the availability of digital health resources does not equate to patients’ ability to use them effectively. For patients who are older, have limited education, or lack experience with digital tools, online health information may be simultaneously supportive and hazardous. On the one hand, online platforms can help patients acquire disease knowledge, medication information, and rehabilitation guidance; on the other hand, unvetted information, commercially driven content, and exaggerated health narratives may increase the risk of misunderstanding, anxiety, and poor decisions. The high proportion of patients in the low eHealth literacy class underscores that, when advancing digital health services for CHD populations, close attention must be paid to the inequities arising from the compounding of the digital divide and the health literacy divide.

The low eHealth literacy group scored significantly higher than the high eHealth literacy group on cyberchondria, psychological inflexibility, and decisional conflict. This finding corroborates the variable-centered results at the person level: patients with lower eHealth literacy not only face informational competence deficits but also exhibit greater health anxiety, more rigid psychological regulation, and more pronounced treatment decision difficulties. Latent classes of eHealth literacy may therefore serve not only as a stratification indicator of digital health capability but also as an important marker for identifying psychological risk and decision-support needs among patients with CHD.

By contrast, the high eHealth literacy group scored lower on cyberchondria, psychological inflexibility, and decisional conflict, suggesting that stronger digital health information competence may confer a degree of psychological protection. Patients with high eHealth literacy are likely better able to identify authoritative sources, comprehend medical terminology, weigh the risks and benefits of treatment alternatives, and integrate online information with clinicians’ recommendations. Such competence may reduce informational uncertainty and perceived threat while enhancing patients’ sense of control over, and participation in, treatment decisions. It should nonetheless be noted that high eHealth literacy does not imply the absence of decisional difficulty or psychological burden. CHD treatment is inherently complex, and even informationally competent patients may experience anxiety and conflict when facing major treatment choices, disease recurrence, or prognostic uncertainty.

### 5.3 Practical and clinical implications

Based on these findings, we recommend incorporating eHealth literacy assessment into routine nursing care and chronic disease management pathways for patients with CHD. Clinicians should evaluate not only whether patients possess CHD-related knowledge but also whether they can effectively search for, understand, discriminate among, and apply online health information. This is especially pertinent when patients are undergoing interventional treatment, establishing long-term medication regimens, engaging in secondary prevention, or choosing among treatment options, as eHealth literacy may directly shape comprehension of treatment information and the quality of decisions. Brief screening instruments could be used to identify patients with low eHealth literacy, who could then be provided with more structured, comprehensible, and actionable informational support.

Whereas traditional health education has emphasized the transfer of disease knowledge, patient education in the digital health era should additionally address information-search strategies, recognition of authoritative sources, appraisal of online information credibility, comprehension of medical risk information, and the ability to discuss online information with clinicians. Health care institutions could provide patients with a curated list of vetted CHD information resources, encouraging preferential use of patient education materials from hospital platforms, professional societies, government health agencies, and evidence-based medicine databases. In outpatient and inpatient education, patients should be encouraged to bring information found online into the clinical encounter, rather than interpreting it and deciding alone. Embedding online information within a professional communication framework can reduce the adverse effects of misinterpretation and anxiety-driven searching on treatment decisions.

For patients who repeatedly search for symptoms online, frequently worry about disease deterioration, focus excessively on treatment risks, or persistently seek reassurance, clinicians should recognize the presence of health anxiety and cyberchondria tendencies. Brief psychoeducation can help patients understand the reciprocal cycle between online searching and anxiety; patients can be advised to limit aimless, repetitive, and nighttime searching, to establish a fixed set of trustworthy information sources, and to seek explanations through formal medical channels when symptoms change or treatment questions arise. For patients whose cyberchondria is severe and impairs daily functioning or treatment adherence, referral to clinical psychology or mental health specialists for further assessment and intervention should be considered.

Uncertainty, fear, and risk trade-offs are unavoidable in CHD treatment decision-making. Patients who attempt to eliminate uncertainty entirely before deciding, or who excessively avoid illness-related emotional experiences, may instead experience greater decisional procrastination and conflict. Intervention approaches grounded in third-wave cognitive-behavioral therapies such as ACT can help patients identify rigid cognitions related to illness and treatment, enhance acceptance of anxiety and uncertainty, and clarify treatment goals in relation to personal values. Such values-oriented psychological support may help patients shift from anxiety-driven repetitive searching and avoidance toward more proactive and stable engagement in treatment.

Finally, the latent profile findings suggest that clinical interventions should be stratified. For the low eHealth literacy group, priority should be given to digital health navigation, recommendation of authoritative resources, emotion management, and decision aids. For the high eHealth literacy group, efforts could focus on further strengthening self-management capacity and clinician–patient collaboration, encouraging active participation in treatment planning on the basis of sound scientific information. Compared with uniform educational programs, profile-based precision interventions are more likely to improve resource efficiency and enhance psychological adaptation and treatment decision quality among patients with CHD.

### 5.4 Limitations and directions for future research

Several limitations warrant consideration. First, the cross-sectional design precludes causal or temporal inference. Although the serial model was theoretically derived, the associations among eHealth literacy, cyberchondria, psychological inflexibility, and decisional conflict are plausibly bidirectional—in particular, cyberchondria and psychological inflexibility may reciprocally reinforce one another, and decisional conflict may itself drive further searching. Longitudinal designs (e.g., cross-lagged panel models or ecological momentary assessment) are needed to disentangle directionality. Second, all variables were self-reported, risking common method, social-desirability, and recall bias; although Harman’s single-factor test was reassuring, it cannot fully rule out systematic measurement error. Future work should incorporate clinician ratings, objective online-behavior logs, and follow-up adherence indicators. Third, although sex, marital status, and revascularization history were controlled, unmeasured confounders—disease severity, comorbidity, depressive symptoms, cognitive function, and the clinician–patient relationship—may remain. Fourth, convenience sampling limits formal generalizability; although the four-province multicenter design improves representativeness, the sample was hospital-based and predominantly older, so findings should be replicated in community-based and younger cohorts. Fifth, the two-profile solution is sample-dependent and requires cross-validation, and its predictive utility for long-term adherence, cardiovascular events, and quality of life remains to be established.

## 6 Conclusion

In patients with CHD, inadequate eHealth literacy is associated with greater decisional conflict, operating largely through a serial pathway of heightened cyberchondria and subsequent psychological inflexibility; eHealth literacy also stratifies into low- and high-literacy subgroups that differ on all three constructs. These findings translate into specific, actionable implications. Clinicians should: (1) screen eHealth literacy (e.g., the 8-item eHEALS) at key decision points—diagnosis, before PCI/CABG, at discharge, and at cardiac-rehabilitation entry—and flag the low-literacy majority for targeted support; (2) provide low-literacy patients with curated, authoritative CHD resources and brief training in appraising online information, and explicitly invite them to bring internet-found content into consultations rather than interpret it alone; (3) identify cyberchondria and deliver brief psychoeducation on the searching–anxiety cycle, with referral to mental-health specialists for severe cases; and (4) integrate brief ACT-based strategies into cardiac rehabilitation to build acceptance of uncertainty and value-guided decision-making. Implementing such profile-based, stepped interventions within routine cardiology care may reduce decisional conflict and improve shared decision-making in the digital health era.

### What’s new

This is among the studies to integrate variable- and person-centered approaches to demonstrate that cyberchondria and psychological inflexibility serially mediate the association between eHealth literacy and decisional conflict in patients with CHD, and to reveal a predominant “low eHealth literacy” latent subgroup that can inform heterogeneity-sensitive decision-support interventions.

## Data Availability

The raw data supporting the conclusions of this article will be made available by the authors, without undue reservation.
